# Coronavirus Disease 2019 (COVID-19) set to increase burden of atherosclerotic cardiovascular disease in Kenya

**DOI:** 10.11604/pamj.supp.2020.35.24762

**Published:** 2020-07-21

**Authors:** Julius Ogeng’o, Paul Bundi Karau, Musa Misiani, Isaac Cheruiyot, Beda Olabu, Brian Ngure Kariuki

**Affiliations:** 1 Department of Human Anatomy, School of Medicine, University of Nairobi, Nairobi, Kenya,; 2 Department of Internal Medicine, School of Medicine, Kenya Methodist University, Meru, Kenya

**Keywords:** COVID-19, cardiovascular disease, atherosclerosis, Kenya, Africa

## Abstract

The coronavirus disease 2019 (COVID-19), first reported in Kenya on March 13, 2020, is spreading rapidly. As of 30th June 2020, over 6,190 cases had been reported with a case fatality of 3.2%. Previous Coronavirus outbreaks have been associated with a significant burden of Cardiovascular disease. For COVID-19, however, there has been no direct reference to potential long-term cardiovascular effects, especially in Africa where atherosclerotic diseases are an emerging challenge. This article, therefore, aims at describing possible long-term effects on the burden of atherosclerotic disease among Kenyans. Available data indicate that COVID-19 and cardiovascular disease share pathomechanisms and risk factors which include ACE2 receptor invasion and renin-angiotensin system signaling, oxidative stress, systemic inflammation, and endothelial dysfunction. Further, SAR-COV-2 infection causes dyslipidemia, dysglycemia, kidney, and liver disease. These mechanisms and diseases constitute risk factors for the initiation, progression, and complications of atherosclerosis. In Kenya, the common risk factors for atherosclerotic cardiovascular disease, and COVID-19 comprising Hypertension, Diabetes Mellitus, Obesity, Cigarette Smoking, Respiratory Tract Infections, Pulmonary Thromboembolism, Chronic Obstructive Pulmonary Disease, and Renal disease are not uncommon and continue to increase. In essence, the prevalence of the common risk factors/comorbidities, between COVID-19 and CVD occurrence of ACE2 receptors on the endothelium, and hence pathomechanisms of SARS-COV-2 infection imply that COVID-19 may increase the burden of atherosclerotic disease in Kenya. All due care should be taken, to prevent and effectively manage the disease, to avert an imminent epidemic of atherosclerotic disease.

## Essay

The novel coronavirus, Severe Acute Respiratory Syndrome Coronavirus 2 (SAR-CoV-2), was reported in December 2019 in Hubei province in China in December 2019 and designated by the World Health Organization as a pandemic in March 2020 [1]. This disease presents perhaps the biggest medical threat in the 21st century. COVID-19 starts as a respiratory tract infection, then attacks other organs and systems that express Angiotensin-Converting Enzyme 2 (ACE-2) receptors such as Cardiovascular, Gastro-Intestinal, and Urinary systems. The disease may then progress into multiorgan and systemic manifestations. Despite the initial predilection of the disease to the lungs, the cardiovascular system appears to have complex interactions with the virus, which has short- and long-term cardiovascular consequences. In Kenya, since the first report on March 20, 2020, the disease has spread rapidly to many parts of the country and in all age groups [2]. The risk factors so far identified include; Cardiovascular Disease, Diabetes Mellitus, Hypertension, Obesity, Chronic Obstructive Pulmonary Disease, Kidney Disease, and Cancer [3]. These diseases are prevalent in Kenya. Africa, including Kenya, has in the recent past been experiencing a surge in atherosclerotic diseases attributable to lifestyle, metabolic and infectious diseases [4]. Whereas the acute Cardiovascular complications of COVID-19 are well documented , the relationship between COVID-19 and long-term CVD complications is seldom acknowledged. Previous Corona Virus outbreaks have been associated with a significant burden of Cardiovascular Disease (CVD). COVID-19 may also have long term complications for cardiovascular disease [3]. Indeed, it is known to accentuate the progression and complications of atherosclerosis [5]. This relationship with cardiovascular disease among Kenyans is, however, not overtly acknowledged. This article highlights some of these implications of COVID-19 for atherosclerotic disease among Kenyans.

COVID-19 reflects the confluence of dysregulated immune-inflammatory response, vascular dysfunction, and thrombosis [6]. It may predispose to atherosclerotic disease through several molecular pathways, metabolic and systemic disorders. The major pathways involved in the pathogenesis of COVID-19 and Cardiovascular disease include ACE 2 receptor invasion and Renin-Angiotensin Signaling; immune dysregulation, inflammation, oxidative stress, endothelial dysfunction, and coagulopathy [7]. These also occur in the CVD risk factors such as Hypertension, Diabetes Mellitus, Chronic obstructive pulmonary disease, Chronic Kidney disease, Acute Respiratory Infection, and cigarette smoking. Coincidentally, these are the same mechanisms, which usually act in concert in the initiation, progression, and complications of atherosclerosis [8]. SARS-COV-2 infects host cells by binding ACE-2 receptors, which are abundant in the blood vessels. The binding of these receptors downregulates ACE-2, causing excessive production of Angiotensin II, and reduced vasodilatory function of Angiotensin 1-7. This process leads to the release of pro-atrophic, pro-fibrotic, pro-inflammatory, and pro-oxidant agents [7]. The virus also binds to sialic acid receptors, Transmembrane serine protease 2 (TMPRSS2), extracellular matrix metalloproteinase inducer (CD147) cathepsin B and L, all expressed in endothelial cells. These receptors facilitate the entry of the virus into the cell [9].

COVID-19 causes oxidative stress consequent to severe hypoxia from acute respiratory damage and high amounts of Reactive Oxygen Species (ROS) [10]. Long-term viral stimulation may elicit an intense immunological reaction, including immune-cell infiltration. Some of the immunocytes like macrophages and neutrophils produce numerous ROS and disruption of antioxidant mechanisms. The high ROS destroys lipids, proteins, and nucleic acid, all of which may result in endothelial dysfunction [7]. Chronic inflammatory disorders are critical risk factors for atherosclerosis and other CVD [11]. SARS-COV-2 elicits the intense release of multiple cytokines and chemokines, in the so-called cytokine storm that leads to vascular inflammation and plaque instability [12]. Some patients may progress to systemic hyper inflammation syndrome in which markers of systemic inflammation are extremely high [13]. The viral invasion also causes the EC to release molecules like ATP, Nuclei acids, ASC oligomers which further trigger the release of proinflammatory cytokines. This upscales a pro-inflammatory feedback loop between EC and monocyte/macrophages. COVID-19 may also define a microvascular injury syndrome mediated by activation of complement pathways [14].

There is substantial evidence that endothelium is the key target in COVID-19. Consequently, endothelial dysfunction may be the hallmark of COVID-19 [9]. SARS-COV-2 infection induces endothelial inflammation, referred to as endothelitis by direct viral invasion and host inflammatory response. Also, it induces apoptosis and pyroptosis [15]. There may also be a direct injury to the endothelium, and mimicry of vasculitis [15]. In the long term, COVID-19, like other SARS-CoV infections causes dyslipidemia, dysglycemia, kidney, and liver diseases in the survivors [16]. These are conditions that are known risk factors for atherosclerosis. Pathological features of atherosclerosis are substantially prevalent in the Kenyan population. Recent studies indicate that over 20% of individuals may display histopathological changes pathognomonic of atherosclerosis in the carotid [17] and coronary [18] arteries. Further, that pre atherosclerotic changes comprising high intima-media thickness are also present in athero-prone vessels [18].

The atherosclerotic diseases in Kenya which are known to constitute risk factors for SARS-COV-2 infection, the severity of symptoms, and signs include coronary artery disease [19], Stroke, and Cerebrovascular disease [20], Pulmonary thromboembolism and hypertensive renal disease [20]. Recent studies indicate that CVD caused over 10% of deaths and 4% of total Disability Adjusted Life Years (DALYs) in 2015, with a steady increase over the last decade [21]. The common risk factors for COVID-19 and CVD include hypertension, diabetes mellitus, and obesity; Pulmonary Thromboembolism; smoking; airway infections and Chronic Obstructive Pulmonary Disease, and Kidney Disease [22]. These conditions are prevalent in Kenya. Hypertension is a common problem in Kenya with a prevalence of 20-25% [23]. It is prevalent even among young people and in rural areas where it was previously thought to be rare. It is more common in men, and urban areas and affects a young population [24]. Poor control of hypertension is associated with dysregulation of the immune system, creating a vicious cycle for severe COVID-19 disease, and more endothelial damage. In Kenya, studies have documented poor control of blood pressure in up to 40% of the affected population, with a significant population being unaware of their hypertensive status [23]. It is, therefore, possible that the occurrence of COVID-19 in hypertensive Kenyans would have long term implications in the cardiovascular system because of the cooperative mechanism of the two conditions and the poor control of blood pressure in the population.

The prevalence of diabetes mellitus and prediabetes is 3-4%, projected to be 4.5% by 2025. Unfortunately, the majority of the patients are not aware of the problem, in many, it is poorly controlled and doctors are yet to adopt universal inquiry about dysglycemia [25]. A study at Kenyatta National Hospital among type 2 diabetic patients documented good glycemic control in only about 37% of the subjects [26]. Obesity is prevalent among diabetic patients, with up to 40% and 80% of diabetic patients in one study having obesity using BMI and Waist-Hip Ratio respectively [26]. The prevalence of overweight and obesity may be as high as >20% [27]. For example, between 2010-2014, over 10M children from 26 SSA countries including Kenya under the age of five (5) years were overweight. It is projected to continue increasing due to unfavorable lifestyle and diet. A study in 2018 found that 20.5% and 9.1% of women in Kenya are overweight and obese respectively [28]. Most cases of obesity are found in urban centers, which coincides with the hotspots for COVID-19. Urbanization and higher socioeconomic status have been linked to an increased prevalence of obesity [27]. Pulmonary thromboembolism is the third leading cause of vascular death after myocardial infarction and stroke [21]. In the Kenyan population, hypertension was found to be one of the commonest comorbidities for pulmonary embolism, occurring in 18.8% of patients, while diabetes occurred in 9.4%. The mean age of thromboembolism victims was 40.8 years, with a peak between 30-50 years. This is incidentally the age-group most affected by COVID-19. This co-occurrence represents a major cardiovascular threat to this population, with worse long-term outcomes.

Smoking is a risk factor for transmission, infection, progression, and adverse outcomes for COVID-19 [29]. The increased susceptibility of smokers to SARS-COV-2 infection has been attributed to decreased immunity and upregulation of ACE-2 receptors. In Kenya, over 10-15% (men much more) of the adult population consume tobacco products. It starts early in life and is sustained, regardless of the awareness of its health hazards. The early commencement coincides with rising prevalence in the working population who are more predisposed to CVD. Two major setbacks are the low level of health workers´ involvement in the cessation of smoking and ignorance of the health hazards of smoking [2]. The vulnerability of smokers to COVID-19 is compounded by the fact that smoking also predisposes to a plethora of respiratory, cardiovascular, gastrointestinal and renal diseases. Consequently, it has the potential to constitute an unfortunate vicious circle between COVID-19 and CVD. Chronic obstructive pulmonary disease (COPD) is estimated to cause disability-adjusted life years (DALYs) similar to Ischemic Heart, disease, stroke, and epilepsy. COPD patients have a 4-5-fold higher risk of COVID-19. The combination of COPD and COVID-19 worsens cardiovascular and another related systematic risk for CVD. Indeed, many studies have reported an increased risk of CVD in patients with acute exacerbation of COPD. Chronic Kidney disease is highly prevalent (30-40%) among Kenyans. It is independently associated with hypertension; obesity; hypertension; diabetes; tobacco smoking factors - lifestyle, dietary; occupation [20]. The link between Kidney disease and COVID-19 is an alteration in ACE2 receptors expressed in the lungs and kidneys. This concurrence of reciprocal risk for COVID-19 and renal disease constitutes another potential threat to long term CVD health.

**Favorable demographic structure:** the age structure of a population is important in the understanding spread and mortality of COVID-19 [30]. Age is a recognized predisposing factor for infection and unfavorable outcomes of COVID-19 in Western and Asian populations [22]. In Kenya, however, the most affected age group is 31-59 years. This age group has better chances of recovery and therefore higher potential to suffer the long-term complications. Coincidentally, the age distribution of COVID-19 resembles the age distribution of cardiovascular disease Myocardial infarction and stroke [19]. It implies that those who are at risk are younger, and will carry the risk for longer. Pertinent to this suggestion is the report that in populations with a high prevalence of obesity, for example, COVID-19 will affect the younger population than hitherto appreciated.

## Conclusion

The diseases which predispose to COVD-19 are already an established, and growing problem among Kenyans. In effect, this implies a substantial number of the population may get infected. These will subsequently spread the virus to otherwise healthy individuals. Consequent to the known pathomechanism, the SARS-COV-2 infection is expected to in the long term increase the burden of Atherosclerotic Cardiovascular Disease. This is a call to awareness and interventional plan.
